# Visuo-proprioceptive integration and recalibration with multiple visual stimuli

**DOI:** 10.1038/s41598-021-00992-2

**Published:** 2021-11-04

**Authors:** Nienke B. Debats, Herbert Heuer, Christoph Kayser

**Affiliations:** 1 Department of Cognitive Neuroscience, Universität Bielefeld, Universitätsstrasse 25, 33615 Bielefeld, Germany; 2 Leibniz Research Centre for Working Environment and Human Factors, Dortmund, Germany; 3 Center for Cognitive Interaction Technology (CITEC), Universität Bielefeld, Bielefeld, Germany

**Keywords:** Sensory processing, Perception

## Abstract

To organize the plethora of sensory signals from our environment into a coherent percept, our brain relies on the processes of multisensory integration and sensory recalibration. We here asked how visuo-proprioceptive integration and recalibration are shaped by the presence of more than one visual stimulus, hence paving the way to study multisensory perception under more naturalistic settings with multiple signals per sensory modality. We used a cursor-control task in which proprioceptive information on the endpoint of a reaching movement was complemented by two visual stimuli providing additional information on the movement endpoint. The visual stimuli were briefly shown, one synchronously with the hand reaching the movement endpoint, the other delayed. In Experiment 1, the judgments of hand movement endpoint revealed integration and recalibration biases oriented towards the position of the synchronous stimulus and away from the delayed one. In Experiment 2 we contrasted two alternative accounts: that only the temporally more proximal visual stimulus enters integration similar to a winner-takes-all process, or that the influences of both stimuli superpose. The proprioceptive biases revealed that integration—and likely also recalibration—are shaped by the superposed contributions of multiple stimuli rather than by only the most powerful individual one.

## Introduction

A key aspect of perception is the combination of redundant multisensory signals, that is, of signals received by separate modalities but relating to the same property of an object. Redundant signals may be discrepant, for example due to sensory noise or environmental distortions such as an echo. Our brain exploits two processes to deal with such discrepancies: multisensory integration and sensory recalibration. Multisensory integration is usually revealed by judgments of bimodal stimuli (e.g.,^1–3^), while recalibration aligns estimates based on unisensory stimuli and is revealed as an aftereffect: a perceptual bias induced by the (prolonged) exposure to bimodal discrepancies (e.g.,^4,5^). While typical laboratory tasks focus on two multisensory stimuli that are combined, real world settings feature an abundance of signals within each modality. This raises the question how multisensory integration and recalibration operate when multiple signals per modality are present.

We investigated the combination of visuo-proprioceptive position information in a cursor-control task: participants made out-and-back movements in a semi-circular workspace with visual feedback provided on a monitor, and subsequently judged the most outward position of their unseen hand (‘movement endpoint’). This task has served as a versatile paradigm to study both integration (e.g.,^6,7^) and recalibration (e.g.,^8–10^). Multisensory binding in this task is facilitated by the experienced coherence of hand and cursor trajectories, even when these are presented in different spatial planes^11^. This experienced coherence suggests a common cause for both signals, which is critical for binding multisensory signals both in this task and in conceptually similar audio-visual (e.g.,^12,13^) and rubber-hand paradigms (e.g.,^14–16^) as well as other variants of multisensory binding (reviews e.g.,^17–19^).

The specific paradigm was designed to probe how estimates of movement endpoints are biased by the presence of two (rather than one) visual stimulus. Because potentially redundant signals are characterized by some form of spatio-temporal proximity, we placed the two visual stimuli with equal spatial proximity to the proprioceptive signal—the hand position as projected in the plane of the monitor—while they featured different temporal proximity to the movement. Two stimuli (each being a cloud of dots) were presented on either side of the movement endpoint on the screen, one synchronously with the hand reaching the endpoint (*Vsynch*), the other with a variable delay after the start of the return movement (*Vdelay*). We contrasted two candidate hypotheses to understand how the temporal proximity of the visual stimuli modulates their influence on integration and recalibration: a ‘winner-takes-all principle’, whereby only one visual stimulus influences proprioceptive judgments of the hand position, and a ‘superposition principle’, whereby the influences of both visual stimuli are summed.

So far, only few studies have investigated multisensory integration or recalibration with multiple stimuli, and these provided mixed results. Importantly, no study directly contrasted the two candidate hypotheses proposed here. In experiments presenting two rubber hands the illusory ownership was experienced for both hands (e.g.,^20,21^), while a bias of the judged hand position was observed for only one of them^22^. In an experiment with two visual stimuli flanking a sound, the sound was mislocalized towards the visual stimulus exhibiting a stronger integration bias when presented as an isolated audio-visual pair^23^, suggesting evidence for the winner-takes all hypothesis though this was not directly tested. Importantly, the multisensory bias in the presence of two stimuli within one modality is not necessarily predictable from that observed for individual stimuli per modality^24,25^, calling for a direct comparison of conditions with one and two candidate stimuli for multisensory binding.

The current study quantified the influence of two visual stimuli on integration and recalibration in the cursor-control paradigm. In Experiment 1 we tested the main hypothesis that the temporally more proximal visual stimulus has a stronger influence on judgments of movement endpoints both during integration and recalibration, and in Experiment 2 we contrasted the two competing hypotheses (winner-takes-all versus superposition) for the integration bias. In each experiment we probed both the biases in the judged position of hand movement endpoints that are relevant to our main hypothesis, and, as an explorative analysis, the biases in the judged position of each visual stimulus. For the former we expected a bias towards the synchronous visual stimulus in bimodal trials, reflecting integration, and in unimodal trials following repeated bimodal exposure, reflecting sensory recalibration. For the latter, we tentatively hypothesized that the position estimates of both visual stimuli are biased toward the movement endpoint, as in conditions with individual visual stimuli these are usually judged towards the position of the hand (e.g.,^6,7,9^).

## Results

### Experiment 1: Integration and recalibration biases with two visual stimuli

**Figure 1:**
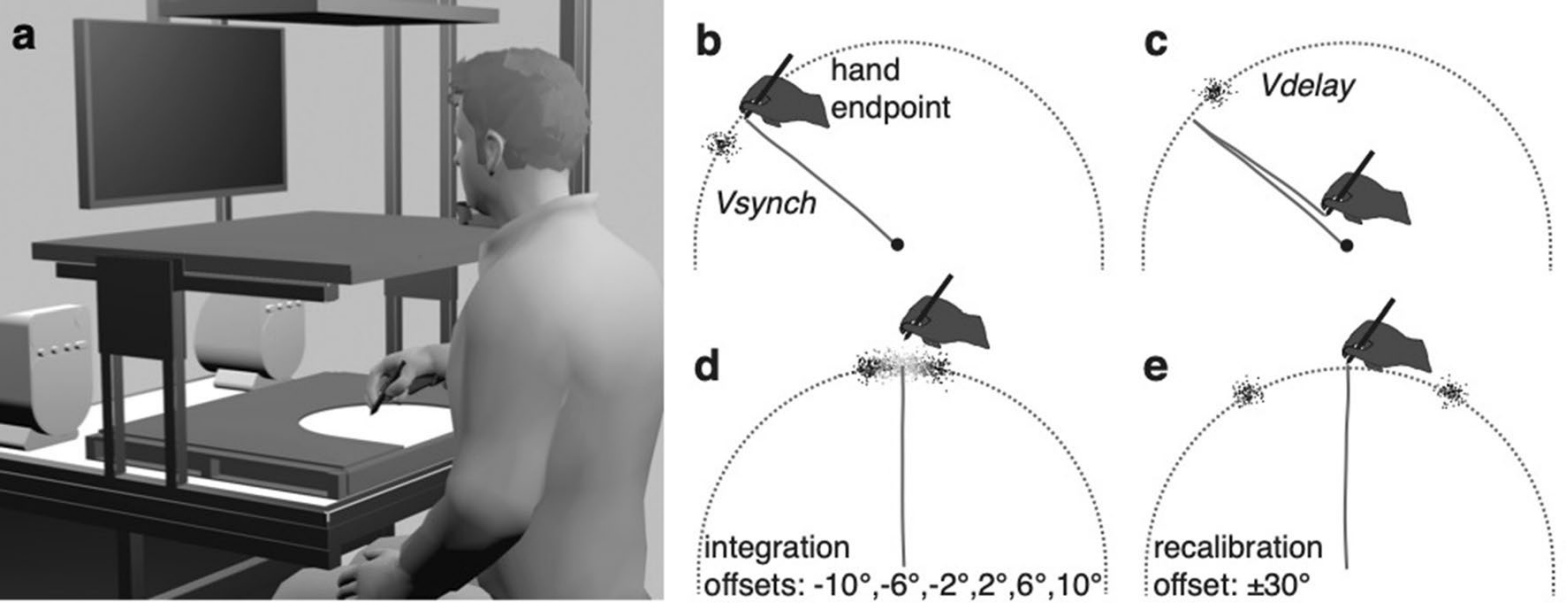
Apparatus, task, and stimuli. (**a**) Participants performed hand movements on a digitizer tablet while visual stimuli were presented on a monitor in front of them. A semi-circular workspace was defined in the horizontal plane by a physical boundary, and in the frontal plane by an invisible semi-circle. In (**b–e**), the two planes are shown on top of each other. (**b**) When the outward-movement ended at the workspace boundary the synchronous visual stimulus was presented (*Vsynch*). Here a visual stimulus with a + 10° angular offset is indicated. (**c**) After the start of the backward-movement towards the workspace’s centre, the delayed visual stimulus (*Vdelay*) was presented around 750 ms later with an angular offset opposite to that of the synchronous stimulus (here: − 10°). (**d**) In the *bimodal judgment* trials used to assess sensory integration, the two visual stimuli had an opposite spatial offset whose magnitude varied from trial to trial (± 10°, ± 6°, or ± 2°). (**e**) In the *bimodal exposure* trials used to induce sensory recalibration, the two visual stimuli had opposite spatial offsets of ± 30° that were constant over trials. Recalibration was assessed in *unimodal judgment* trials, in which only one stimulus was present (hand, *Vsynch*, or *Vdelay*).

In Experiment 1 we asked how visuo-proprioceptive integration and recalibration are affected by the presence of two visual stimuli. Twenty participants performed out-and-back hand movements whereby visual stimuli could be presented on a monitor, their position being related to the endpoint of the hand movement as mapped onto the monitor frame of reference (see Fig. 1a). Each visual stimulus was a Gaussian cloud of 100 small dots, coloured either blue or yellow, and presented for 100 ms. One stimulus was presented synchronously with the hand reaching the movement endpoint (*Vsynch*; see Fig. 1b), the other was delayed (*Vdelay*; see Fig. 1c). *Vsynch* and *Vdelay* were presented with opposite spatial offsets relative to the movement endpoint, as illustrated in Fig. 1b, c; for instance, if *Vsynch* had an offset of − 6°, *Vdelay* would follow with + 6° offset. Upon completing the return movement, participants judged the position of the movement endpoint, the “blue cloud”, or the “yellow cloud”, by placing a single white dot on the monitor in the appropriate location along an invisible semi-circular track running through all possible positions.

#### Integration biases

**Figure 2:**
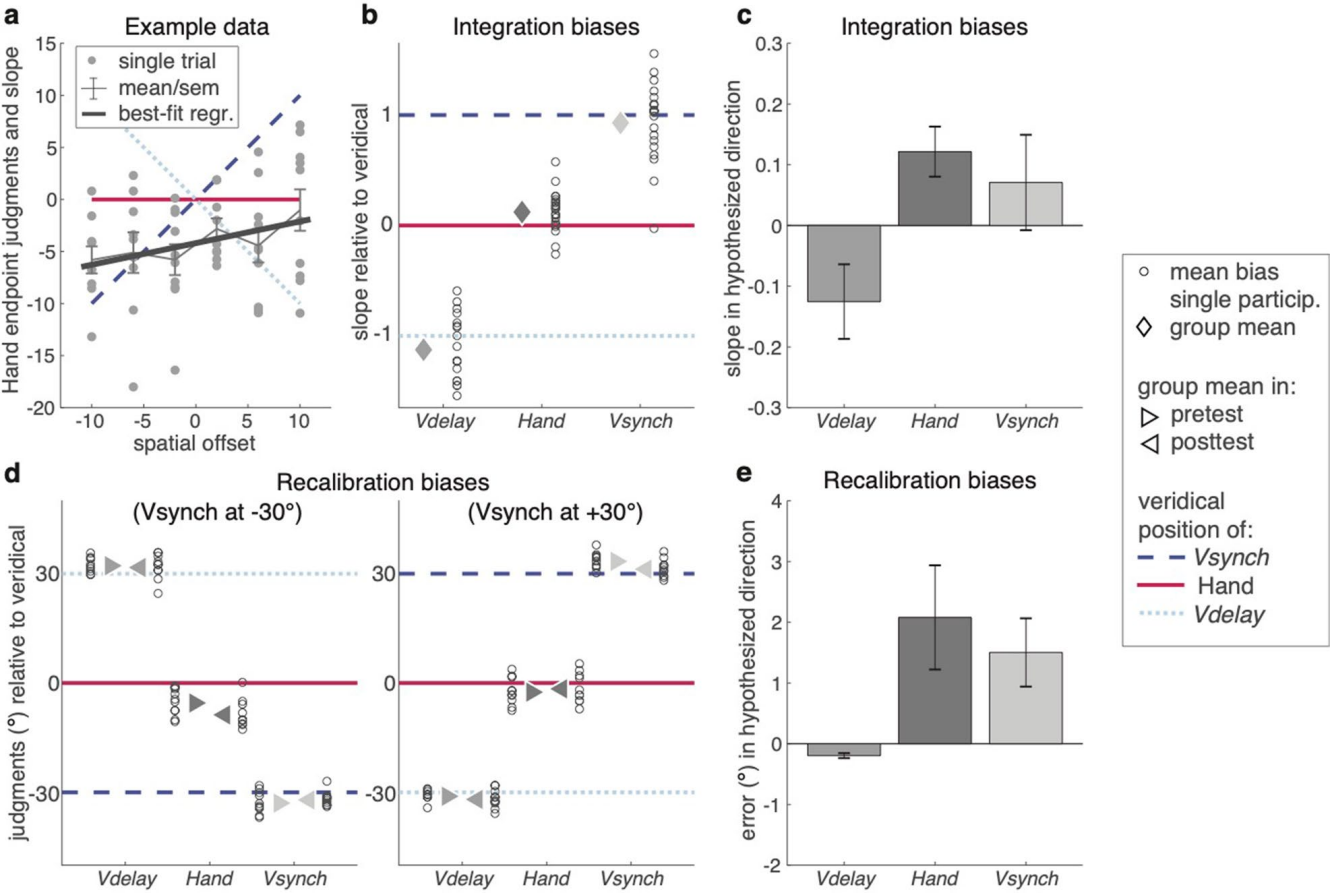
Integration and recalibration biases in experiment 1: In all panels, horizontal solid red lines indicate the veridical hand endpoint, the diagonal broken blue line that of *Vsynch*, and the diagonal dotted light blue line that of *Vdelay*. (**a**) Integration biases were derived as slopes of linear regressions. This panel shows an example of judgments of the hand endpoints in *bimodal judgment* trials. The grey dots mark the judgment errors in individual trials. For this participant, there was a positive slope of .21, indicating that the judgments of hand endpoints were biased towards the position of *Vsynch*. (**b**) Individual participants’ integration biases relative to the veridical stimulus positions. The small open circles indicate individual participants’ mean slopes; diamonds indicate the group mean. (**c**) The average integration biases (mean ± SEM) in the hypothesized direction: Hand endpoint towards *Vsynch*, *Vsynch* and *Vdelay* towards the hand endpoint. (**d**) Recalibration biases (judgment errors in degrees) for the pre-test and the post-test relative to the veridical positions of the hand, *Vsynch*, and *Vdelay*. The results are shown separately for the participants that received *Vsynch* at − 30° or at + 30° offset. (**e**) The average recalibration biases (mean ± SEM) as differences between post-test and pre-test with the positive sign indicating the expected direction of the bias: hand endpoint towards where *Vsynch* had been presented in the preceding *exposure* trials, *Vsynch* and *Vdelay* towards where the hand endpoint had been in the preceding *exposure* trials.

 Integration was tested across six spatial offsets of *Vsynch* relative to the movement endpoint (− 10°, − 6°, − 2°, 2°, 6° and 10°; Fig. 1d). Participants judged the position of the movement endpoint in 60 trials, and the integration bias (toward the position of either *Vsynch* or *Vdelay*) was obtained as the regression slope of the trial-wise judgment errors against the respective spatial offsets (Fig. 2a; black line). A positive slope indicates a bias towards *Vsynch*. Similar regression analyses were used to obtain the biases in the judged positions of *Vsynch* and *Vdelay* relative to the movement endpoint (60 trials each). Figure 2b shows all three integration biases with the sign matching the physical stimulus offsets; Fig. 2c shows the biases with positive values corresponding to the hypothesized directions: proprioceptive bias towards *Vsynch* and visual biases towards the hand.

Concerning our main hypothesis, judgments of the movement endpoint were significantly biased towards *Vsynch* (*t*(19) = 2.88, Cohen’s d_z_ = 0.644, *p* = 0.010). This bias amounted to 12.2% of the spatial offset and pointed away from the position of *Vdelay*. In contrast and relating to the more explorative analyses, judgments of *Vsynch* were not significantly biased (*t*(19) = 0.88, d_z_ = 0.197, *p* = 0.390), and judgments of *Vdelay* were biased away from the hand position, though not significantly (*t*(19) = 1.99, d_z_ = 0.445, *p* = 0.061).

We complemented these main results by additional checks. The intercepts of the regression lines reflect overall judgment biases independent of spatial offsets. For the hand position, this bias was clockwise (mean ± SEM: − 3.23° ± 0.53°; *t*(19) = 5.90, d_z_ = 1.320, *p* < 0.001), whereas there were no significant overall biases for *Vsynch* (− 0.42° ± 0.25°; *t*(19) = 1.65, d_z_ = 0.369, *p* = 0.116) or *Vdelay* (− 0.53° ± 0.29°; *t*(19) = 1.85, d_z_ = 0.413, *p* = 0.080). Second, we quantified intra-individual judgment variability as the standard deviation of the residuals of the regression. Variability differed between cued judgments (*F*(2,38) = 7.71, ε = 0.96, $\eta^{2}$ = 0.289, *p* = 0.002): it was largest for *Vsynch* (7.22° ± 0.29°) and smaller for *Vdelay* (6.41° ± 0.29°) and the hand (6.45° ± 0.32°). Third, we scrutinized the durations of various within-trial intervals and found no differences between cued judgments that could point to potential confounding effects of temporal delays. The duration from the start signal for the movement until *Vsynch* varied between 1.42 and 1.43 s across cued judgments (*F*(2,38) = 0.17, ε = 0.96, $\eta_{p}^{2}$ = 0.009, *p* = 0.842), until *Vdelay* between 2.48 and 2.50 s (*F*(2,38) = 0.39, ε = 0.98, $\eta_{p}^{2}$ = 0.020, *p* = 0.682), and until the judgment instruction between 3.02 and 3.04 s (*F*(2,38) = 0.33, ε = 0.88, $\eta_{p}^{2}$ = 0.017, *p* = 0.723); the time between judgment instruction and end of the judgment varied between 6.23 and 6.25 s (*F*(2,38) = 0.005, ε = 0.99, $\eta_{p}^{2}$ < 0.001, *p* = 0.995).

#### Recalibration biases

Recalibration blocks consisted of a pre-test and a post-test phase, separated by an exposure phase. The exposure consisted of 180 bimodal trials with *Vsynch* and *Vdelay* presented at constant spatial offsets of − 30° and + 30° (see Fig. 1e). The pre-test and post-test contained 30 unimodal trials, 10 trials each with only the hand movement, only *Vsynch*, or only *Vdelay*. Figure 2d illustrates the mean judgment errors in these pre-test and post-test trials; the coloured lines indicate the relative stimulus positions as they were during exposure. The two graphs show the data separately for participants with spatial offsets of *Vsynch* at − 30° and + 30°, respectively. Figure 2e shows the recalibration biases, the changes of judgment errors from pre-test to post-test, such that positive values reflect a bias in the expected direction.

Concerning our main hypothesis, proprioceptive recalibration (shift of the movement-endpoint judgments from pre-test to post-test) was towards *Vsynch* (*t*(19) = 2.36, d_z_ = 0.529, *p* = 0.029) and thus away from *Vdelay*. For the explorative analyses, we found evidence of visual recalibration, as judgments of *Vsynch* were shifted toward the hand position in the preceding exposure trials (*t*(19) = 2.61, d_z_ = 0.583, *p* = 0.017); judgments of *Vdelay* did not differ between pre- and post-test (*t*(19) = 0.38, d_z_ = 0.085, *p* = 0.708).

In addition to recalibration, we probed for signs of motor adaptation, which typically accompanies proprioceptive recalibration. It results in an adaptive shift of the direction of hand movements from pre-test to post-test which partially compensates the spatial offset between visual stimulus and hand (e.g.,^26–28^). Here motor adaptation (quantified as the shift of movement direction from pre-test to post-test toward *Vsynch* in the preceding exposure trials) was small and not significant (1.46° ± 1.34°; *t*(19) = 1.09, d_z_ = 0.244, *p* = 0.288). This result is inconsistent with previous observations that motor adaptation is generally stronger than sensory recalibration, yet consistent with the notion that motor adaptation and proprioceptive recalibration are partially independent (cf.^9,29–31^).

As for integration, we ran additional checks. First, judgment errors in the pre-test prior to recalibration were in the clockwise direction for the hand position (− 3.99° ± 0.85°; *t*(19) =  − 4.60, d_z_ = 1.028, *p* < 0.001; Fig. 2d), but absent for *Vsynch* or *Vdelay* (0.22° ± 0.90° and 0.56° ± 0.53°). Second, the intra-individual judgment variability differed between unimodal trial types (*F*(2,38) = 5.73, ε = 0.98, $\eta_{p}^{2}$ = 0.232, *p* = 0.007), but not between pre- and post-test (*F*(1,19) = 0.83, $\eta_{p}^{2}$ = 042, *p* = 0.375), and there was no significant interaction (*F*(2,38) = 1.76, ε = 0.97, $\eta_{p}^{2}$ = 0.085, *p* = 0.187). Judgment variability was largest for the hand (7.69° ± 0.51°) and smaller for *Vsynch* (5.99° ± 0.44°) and *Vdelay* (6.06° ± 0.43°). Third, the durations between start signal and judgment instruction in unimodal test trials ranged between 2.8 and 2.9 s. None of the main effects was statistically significant (trial type: *F*(2,38) = 1.45, ε = 0.77, $\eta_{p}^{2}$ = 0.071, *p* = 0.248; test: *F*(1,19) = 3.05, $\eta_{p}^{2}$ = 0.138, *p* = 0.097), neither was the interaction (*F*(2,38) = 1.41, ε = 0.69,$\eta_{p}^{2}$ = 0.069, *p* = 0.256). Judgment durations were longest for *Vdelay* (7.03 ± 0.40 s), shorter for *Vsynch* (6.27 ± 0.23 s), and shortest for the hand (5.62 ± 0.17 s). These differences were statistically significant (*F*(2,38) = 8.80, ε = 0.79, $\eta_{p}^{2}$ = 0.317, *p* = 0.002), whereas neither the difference between pre-test and post-test (6.28 ± 0.25 s versus 6.34 ± 0.22 s; *F*(1,19) = 0.06,$\eta_{p}^{2}$ = 0.003, *p* = 0.808) nor the interaction (*F*(2,38) = 1.35, ε = 0.96, $\eta_{p}^{2}$ = 0.066, *p* = 0.271) were significant. Thus, there were no substantial differences in the within-trial timing between pre-test and post-test that could have affected the observed recalibration biases.

### Experiment 2: Winner-takes-all versus superposition hypothesis

Experiment 2 was designed to arbitrate between the winner-takes-all hypothesis, according to which only the temporally most proximal visual signal enters integration and recalibration, and the superposition hypothesis, according to which the biases induced by both visual signals are summed. We compared a condition in which both visual stimuli were presented (*double-stimulus* condition) with conditions in which only one of them was presented (*synchronous-stimulus* and *delayed-stimulus* conditions). Specifically, we tested whether the bias of the judged hand position towards *Vsynch* was equivalent in the presence and absence of *Vdelay* (in line with the winner-takes-all hypothesis), or whether the bias in the presence of two visual stimuli matched the sum of the biases induced by each of the visual stimuli alone (in line with the superposition hypothesis). For reasons of economy, we confined ourselves to multisensory integration, where the within-participant comparison of the different conditions with one or two visual stimuli is feasible. The equivalent test for recalibration would require three different participant groups who adapt to opposite spatial offsets of two visual stimuli and to the spatial offsets of the individual stimuli.

**Figure 3:**
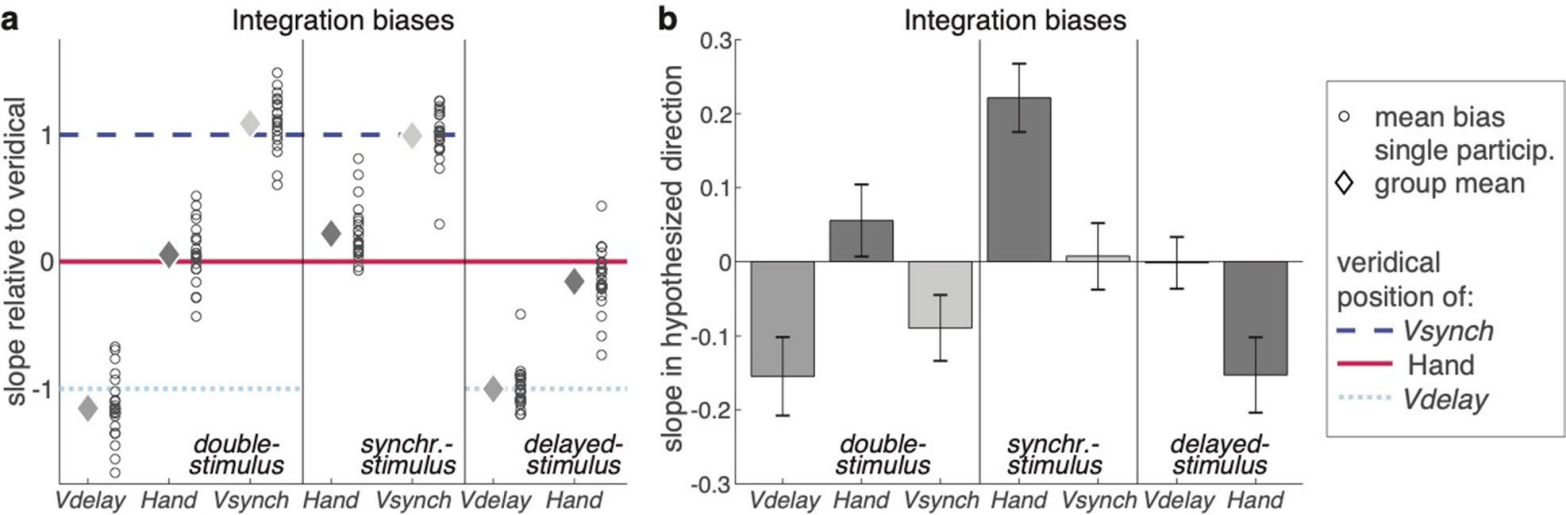
Integration biases in Experiment 2. (**a**) Integration biases (i.e., slopes of the regression analyses) relative to the veridical positions of the hand endpoints (solid red line), of *Vdelay* (dotted light blue line), and of *Vsynch* (broken blue line) for double-stimulus, synchronous-stimulus, and delayed-stimulus trials. The open circles illustrate individual participants’ bias in the respective trials; the diamonds illustrate the group mean. (**b**) The average integration biases (mean ± SEM) in the hypothesized directions: hand endpoint towards *Vsynch, Vsynch and Vdelay* towards the hand endpoint. For the delayed-stimulus trials this means that the negative bias of the hand-position judgments indicates a bias away from the invisible *Vsynch* and towards the visible *Vdelay*.

Figure 3a illustrates the integration biases for each judgment (hand, *Vsynch*, *Vdelay*) relative to the physical stimulus position. Figure 3b displays the mean proportional biases towards the hypothesized directions: positive biases for the hand are towards *Vsynch* and positive biases for *Vsynch* and *Vdelay* are towards the hand. With this convention the proportional bias of the judged hand position towards the position of *Vdelay* in the *delayed-stimulus* condition should be negative rather than positive, that is, away from the position of (the absent) *Vsynch*.

In the *double-stimulus* trials, the bias of the judged hand position was positive, hence towards *Vsynch* and away from *Vdelay*. Different from Experiment 1, the bias was not significantly different from zero (*t*(21) = 1.11, d_z_ = 0.237, *p* = 0.278), but the difference between experiments was not significant either (*t*(40) = 1.00, d = 0.309, *p* = 0.323). When the data of both experiments were combined, the proprioceptive bias was significant (0.087 ± 0.033; *t*(41) = 2.64, d_z_ = 0.407, *p* = 0.012). In *synchronous-stimulus* trials, the bias of the judged hand position towards *Vsynch* was significant (*t*(21) = 4.68, d_z_ = 0.998, *p* < 0.001), and in *delayed-stimulus* trials, the bias was negative and significant (*t*(21) = 2.93, d_z_ = 0.624, *p* = 0.008), hence away from the position of (the not presented) *Vsynch* and towards the position of *Vdelay*.

Under the winner-takes-all hypothesis, the bias of the judged hand position towards *Vsynch* should be the same in *double-stimulus* and *synchronous-stimulus* trials, whereas under the superposition hypothesis it should be larger in *synchronous-stimulus* trials: testing our main hypothesis here, we found that the latter was the case (*t*(21) = 2.96, d_z_ = 0.632, *p* = 0.007). Further, under the winner-takes-all hypothesis, the sum of the biases in *synchronous-stimulus* and *delayed-stimulus* trials should be smaller than the bias towards *Vsynch* in *double-stimulus* trials, whereas under the superposition hypothesis it should be the same. In our data the sum was 0.069 ± 0.051 and not significantly different from the bias in *double-stimulus* trials, which was 0.056 ± 0.050 (*t*(21) = 0.22, d_z_ = 0.047, *p* = 0.829). Thus, the data are consistent with the superposition hypothesis and at variance with the winner-takes-all hypothesis.

For the more explorative analyses, we found that the visual biases for the judgments of *Vsynch* and *Vdelay* in *double-stimulus* trials were comparable to those in Experiment 1. For *Vsynch*, the bias did not differ significantly from Experiment 1 (*t*(40) = 1.77, d = 0.547, *p* = 0.085), and for the combined sample it did not differ from zero (− 0.012 ± 0.046 (*t*(41) = 0.280, d_z_ = 0.043, *p* = 0.781). For *Vdelay*, the bias did also not differ significantly from Experiment 1 (*t*(40) = 0.35, d = 0.109, *p* = 0.726), but for the combined sample it was significant (− 0.141 ± 0.041; *t*(41) = 3.440, d_z_ = 0.531, *p* = 0.001), pointing away from the hand. Thus, across both experiments, there was no reliable integration bias for *Vsynch* and a reliable repulsion for *Vdelay*. The visual biases in the *synchronous-stimulus* and the *delayed-stimulus* trials were close to zero (cf. Figure 3). For *Vsynch* the difference between *double-stimulus* and *synchronous-stimulus* trials did not reach significance (*t*(21) = 1.84, d_z_ = 0.392, *p* = 0.080), whereas for *Vdelay* the difference between *double-stimulus* and *delayed-stimulus* trials was significant (t(21) = 2.65, d_z_ = 0.565, p = 0.015). Thus, the repulsion of *Vdelay* in *double-stimulus* trials depended on the preceding presentation of *Vsynch*, implying that the repulsion was not away from the position of the hand, but from the position of *Vsynch*.

Finally, the overall biases and the intra-individual variabilities were comparable to Experiment 1. The overall bias for hand judgments, averaged across trial types, was clockwise (− 3.18 ± 0.71°), while no consistent biases emerged for the visual stimuli (*Vsynch*: − 0.06 ± 0.28°; *Vdelay*: − 0.12 ± 0.28°). Judgment variability in *double-stimulus* trials differed according to cued judgment (*F*(2,42) = 11.17, ε = 0.92, $\eta_{p}^{2}$ = 0.347, *p* < 0.001); as in Experiment 1, variability was largest for *Vsynch* (8.32° ± 0.37°) and smaller for *Vdelay* (6.81° ± 0.28°) or the hand (7.22 ± 0.35°). Also, as in Experiment 1, the analysis of the various within-trial durations revealed no systematic variations across trial types that could have affected the main findings. The duration from the signal to start the movement until *Vsynch* was 1.24 ± 0.04 s in *double-stimulus* and 1.25 ± 0.04 s in *synchronous-stimulus* trials (*t*(21) = 1.45, d_z_ = 0.309, *p* = 0.162), and until *Vdelay* it was 2.22 ± 0.05 s in *double-stimulus* and 2.23 ± 0.05 s in *delayed-stimulus trials* (*t*(21) = 0.58, d_z_ = 0.124, *p* = 0.566). The time to the judgment instruction was 2.64 ± 0.07 s, 2.67 ± 0.07 s, and 2.66 ± 0.07 s in *double-stimulus*, *synchronous-stimulus*, and *delayed-stimulus* trials, respectively, (*F*(2,42) = 1.48, ε = 0.81, $\eta_{p}^{2}$ = 0.065, *p* = 0.242 ), and the judgment duration was 5.80 ± 0.21 s, 6.00 ± 0.24 s, and 5.77 ± 0.21 s in the three types of trials (*F*(2,42) = 3.10, ε = 0.95, $\eta_{p}^{2}$ = 0.129, *p* = 0.058).

## Discussion

Multisensory integration and recalibration are two processes by which our brain exploits multisensory information. Integration reduces discrepancies between two redundant signals, while recalibration reduces consistent discrepancies by aligning the unisensory estimates. Importantly, for multisensory perception to function in every day settings, our brain has to first identify the relevant redundant signals for integration and recalibration (e.g.,^17–19^). Here we asked how two stimuli in one modality are combined with evidence in another modality. We found that when two visual stimuli provide potential information about movement endpoints in addition to the proprioceptive feedback, the judged endpoints of the hand movement are biased towards the position of the synchronous visual stimulus and away from the delayed one, both in bimodal trials (integration) and in unimodal trials subsequent to a series of bimodal ones (recalibration). Hence, the synchronous visual signal dominates integration and recalibration. This dominance could in principle result from a winner-takes-all interaction between the visual stimuli, where only the dominant one attracts the perceived position of the hand, or from the superposition of two biases, where the stronger attraction by the synchronous visual stimulus is superposed on the weaker attraction by the delayed one. When contrasting these hypotheses for integration we found support for the superposition hypothesis. Thus, judgments of the hand position are influenced by both visual stimuli, though the temporally more proximal one exerts a stronger influence.

We propose that the superposition hypothesis similarly holds for recalibration, for a number of reasons. First, the proprioceptive recalibration bias with two visual stimuli amounted to only about 7% of the visuo-proprioceptive offset, whereas about 20% are typical with single visual stimuli^8,9,31^. Paralleling this, the proprioceptive integration bias with two visual stimuli amounted to only about 9% and was clearly smaller than the 22% or more observed with the single synchronous visual stimulus both in experiment 2 and previously^32^. This concordance suggests that the delayed visual stimuli influenced both integration and recalibration. Second, integration and recalibration bear functional similarities: both are responses to redundant multimodal signals, whereby one is the immediate effect while the other is the respective aftereffect. Finally, both are governed by the reliability rule (e.g.,^7,33^), are supposedly steered by perceptual causal inference (e.g.,^34–36^), and are supposedly supported by shared brain regions^37,38^.

### Temporal proximity shapes multisensory binding

The dominance of the synchronous stimulus rests in the general relevance of temporal proximity for multisensory binding. Temporal proximity shapes explicit and implicit judgments of sensory redundancy and of causal relations (e.g.,^39,40^), two key drivers of multisensory binding (reviews e.g.,^17–19^). Our results hence suggest that the synchronous visual stimulus was perceived as more causally related to the movement endpoint.

One may argue that differences in temporal offset between the stimuli and the actual judgment at the end of the trial contributed to this effect, as this delay was about 1.5 s for the synchronous and about 0.5 s for the delayed visual stimulus. Yet, we consider this as a minor concern. First, a one-second difference is only small in relation to the duration of the judgment itself, which was about 6 s. Second, a recent study on audio-visual integration and recalibration suggests that adding a temporal delay between stimuli and responses has negligible effects^41^. Thus, the relevant temporal offset that affects integration and recalibration should be the one between stimuli, not the one between stimuli and judgments.

### Judgment biases of visual stimuli

As explorative analyses, we also investigated the judgment biases of the visual stimuli. While these were overall small, the integration bias of the delayed visual stimulus exhibited a remarkable repulsion away from the movement endpoint, and thus also from the synchronous visual stimulus. Such a repulsion was absent in trials without the synchronous stimulus (*delayed-stimulus trials* in Experiment 2), suggesting that the delayed visual stimulus was repulsed by the other visual stimulus rather than the proprioceptive signal. For recalibration we observed no such repulsion, suggesting that the repulsive effect requires the presence of the synchronous stimulus in the same trial.

We envisage different factors contributing to this repulsion, starting with gaze direction. At the time the delayed visual stimulus was presented, participants may still have attended or fixated the position of the synchronous visual stimulus. Thus, the distance of the delayed stimulus from the synchronous one may have been overestimated because of its retinal eccentricity^42,43^, and the perceived position of the delayed stimulus may have been subject to an attentional repulsion effect (e.g.,^44,45^). In addition, there might be a more generic mechanism responsible for the repulsion of the delayed visual stimulus. There is a parallel between the spatial attraction of an action (i.e., movement endpoint) and its putative visual consequence, and the temporal attraction between an action (e.g. keypress) and its auditory effect—a phenomenon known as ‘intentional binding’^46,47^. Such binding has been observed with single auditory stimuli following or preceding a keypress, whereas for two successive stimuli or keypresses attraction was absent or turned into repulsion^48^. Combined, these previous and current findings may hint at a general repulsive effect for the second-in-line sensory consequence of an action, both in space and time.

We observed no reliable integration biases for the individually-presented visual stimuli in Experiment 2. Although such visual biases are generally small^7,11,36,49,50^, their absence was still unexpected but may be explained by two unique features distinguishing Experiment 2 from previous studies: first, the clouds of dots were less salient than the single dots used previously, which may render the respective judgments more variable, and second, Experiment 2 mixed trials with one or two visual stimuli in each block. This may have reduced the experienced redundancy between visual and proprioceptive information and thereby the integration biases.

### The role of spatial discrepancies in multisensory binding

Similar to other frequently studied multisensory paradigms such as the audio-visual ventriloquist (e.g.,^51^) or rubber-hand paradigms (e.g.,^14,52–55^), multisensory binding in the present task is facilitated by the experienced coherence of stimuli and serves to reduce apparent spatial discrepancies between sensory signals that are perceived as being causally related. Importantly, multisensory binding here arises despite hand and cursor being spatially offset along two dimensions: the angular offset between the movement endpoint and the visual signals on the screen, and the much larger spatial offset that exists because the actual movement and visual signals reside in distinct spatial planes. The persistence of multisensory binding despite the large spatial separation is a feature that distinguishes the cursor-control task from the other paradigms^11^ and allows insights not immediately evident from these alone. For example, both the cursor-control and the rubber-hand paradigm are visuo-proprioceptive tasks, but in the rubber-hand paradigm binding of the signals is more or less restricted to the immediate peripersonal space^56^ and often to visual stimuli with similar shape as the hand^57^. The cursor-control paradigm reveals the existence of conditions under which both these constraints on binding are absent.

## Conclusion

We investigated multisensory binding in settings with more than one pair of possibly redundant cross-modal stimuli, a step towards more complex natural situations where redundant signals for integration and recalibration have to be identified among a plethora of signals. We found that the use of redundant stimuli for multisensory integration follows a superposition principle and propose that this principle also holds for recalibration, a hypothesis to be tested in future studies.

## Methods

### Participants

We collected data from 20 participants for Experiment 1 (aged 18 to 41 years, mean ± sd: 25.3 ± 5.9 years; 15 female) and 22 participants for Experiment 2 (aged 19 to 30 years; mean ± sd: 24.5 ± 2.9 years; 10 female). According to G*Power 3.1.9.2^58^, this sample-size is sufficient to detect medium-sized integration and recalibration effects with an error probability of 0.05 and power of 0.8. (Effect size d_z_ was set to 0.6, which is about half the effect size computed from the integration biases found by Debats and Heuer^32^ and less than half the recalibration biases reported by Rand and Heuer^9^ for experimental paradigms similar to the present one.) In Experiment 1, the data of five additional participants was replaced because after data screening only few valid trials remained (see below). All participants were right handed according to a German version of the Edinburgh handedness inventory^59^. The experiments were conducted in accordance with the declaration of Helsinki and approved by the Bielefeld University Ethics Committee. Participants gave written informed consent prior to participation and were compensated with a payment of €7 per hour.

### Apparatus

Participants sat in front of a table with their head stabilized by a chinrest. A digitizer tablet (Wacom Intuos4 XL; 48.8 by 30.5 cm) was placed on the table, and a computer monitor (Samsung MD230; 23 inches; 50.9 by 28.6 cm) was mounted at 60 cm viewing distance (Fig. 1a). Participants held a stylus with their right hand and pressed a button on this when required to submit responses. The stylus could be moved on the digitizer within a semi-circular workspace of 15 cm radius that was bounded by a 5 mm thick PVC template. The boundary served as a mechanical stop for outward movements. A horizontal opaque board prevented direct vision of hand and stylus. The position of the stylus was recorded at 60 Hz with a spatial resolution of 0.01 mm, using a custom-built MATLAB program with the Psychophysics Toolbox^60^. During the experiment the room lights were off. All stimuli on the monitor were presented in light grey on a black background with the exceptions described below.

### Task

**Figure 4:**
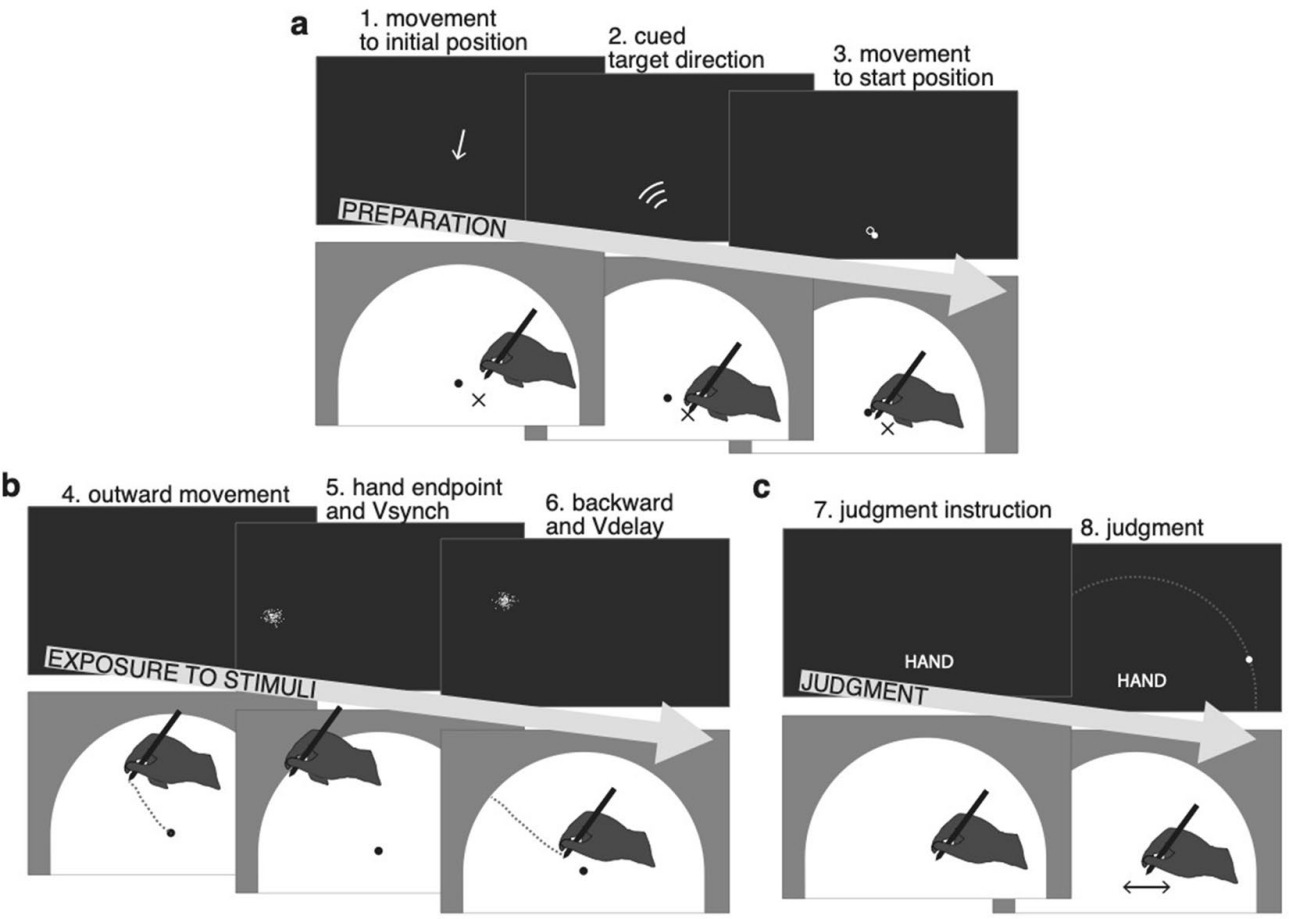
Experimental trials. (**a**) During the preparation phase of each trial, participants were guided by arrows on the monitor to an initial position, and a direction for the outward movement was cued. Participants then moved the cursor to the start position in the centre of the workspace. (**b**) During the out-and-back movement, different stimuli could be presented. Depending on trial type, this could be the *hand endpoint* (i.e., the position of the tip of the stylus when hitting the workspace boundary), and/or the visual stimulus (cloud of dots) synchronous with the hand reaching the workspace boundary (*Vsynch*), and/or the delayed visual stimulus, presented around 750 ms after starting the backward movement (*Vdelay*). One of the visual stimuli was blue, the other yellow. Both were located on the invisible semi-circle that corresponded to the workspace boundary. (**c**) In trials that included a position judgment, a written instruction was shown on the monitor to indicate which position was to be judged: the hand endpoint, the (centre of the) blue cloud, or the (centre of the) yellow cloud. Judgments were indicated on the monitor by means of steering a small dot to the desired position on the semi-circular track that corresponded to the workspace boundary (the track is shown here for reasons of clarity, but was not visible to the participants).

A general task-description is provided here, with more details below and in Fig. 4. Participants made out-and-back movement with their (unseen) right hand; each movement proceeded from the centre of the semi-circular workspace to its boundary and back to the centre. Most relevant was the endpoint of the outward movement (i.e., where the hand-held stylus touched the boundary). To prevent stereotypical endpoints, an approximate direction for the hand movement was cued in each trial, centred at one of five possible angles (60°, 75°, 90° (straight ahead), 105°, or 120°). Two visual stimuli on the monitor (clouds of dots) provided visual information on the endpoint. One visual stimulus appeared when the hand reached the workspace boundary (the synchronous visual stimulus: *Vsynch*; Fig. 1b), and the other was delayed such that it appeared during the backward movement (the delayed visual stimulus: *Vdelay*; Fig. 1c). The visual stimuli had opposite spatial offsets from the movement endpoint. The offset is necessary to reveal integration and to induce recalibration.

After completion of the backward movement, participants were cued to indicate either the perceived movement endpoint or the position of one of the two visual stimuli. They did so by moving a small white dot on the monitor along an invisible semi-circular track that corresponded to the workspace boundary*.* Judgments of hand endpoints were thus cross-modal (proprioceptive to visual) and required a reference-frame transformation (horizontal plane to fronto-parallel plane), whereas judgments of the positions of the visual stimuli were intra-modal and required no such transformation. According to previous work^11^, the different frames of reference have only marginal or no effects on the strength of integration. Yet cross-modal judgments are noisier than intra-modal judgments and therefore affect the asymmetry of the biases^7^.

### Visual stimuli

Visual stimuli were clouds of 100 dots of 1 mm diameter each. Their positions had a bivariate normal distribution with a standard deviation (SD) of 5 mm. Dots that deviated by more than ± 3 SD from the centre on one of the coordinates were moved into this range. These clouds effectively had a diameter of 30 mm (corresponding to 2.86° of visual angle, or 11.46° in the polar coordinates). The bivariate normal distribution was centred on the polar coordinates (α + ρ, r), with r the radius of the invisible semi-circle (150 mm), α the angular position of the movement endpoint, and ρ the angular spatial offset between visual stimulus and movement endpoint (positive ρ was counter clockwise, negative ρ was clockwise). For testing integration, we used small and randomly varying angular spatial offsets (− 10°, − 6°, − 2°, 2°, 6°, 10°; Fig. 1d); for inducing recalibration we used larger consistent offsets (+ 30° and − 30°; Fig. 1e).

The synchronous visual stimulus was presented for 100 ms when the stylus passed a threshold of 97% of the workspace radius from the centre. This 97% criterion was just below the movement distance at which the stylus would hit the boundary. The delayed stimulus was presented for 100 ms with a normally distributed delay (mean: 750 ms, SD: 80 ms) after the backward movement had passed the 97% threshold. A degree of variability was added to the delay to enhance the temporal inconsistency between the movement endpoint and the delayed visual stimulus.

Visual stimuli were either presented in blue (RGB: 0 155 155) or yellow (RGB: 155 155 0); for half of the participants the synchronous stimulus was blue and the delayed one was yellow, for the other half this was reversed. The different colours were used to facilitate judgment instruction (‘blue cloud’ or ‘yellow cloud’).

### Trial types

Experiment 1 comprised three types of trials that differed with respect to the stimuli presented (hand, *Vsynch*, *Vdelay*) and the judgment required (hand, *Vsynch*, *Vdelay*, none). In *bimodal judgment trials*, all three stimuli were presented and the position of one of them had to be judged. In *bimodal exposure* trials, all three stimuli were presented, but no judgment was required. In *unimodal judgment* trials, only one out of the three stimuli was presented and its position judged. When a visual stimulus was presented without a movement being performed, the terms ‘synchronous’ and ‘delayed’ refer to shorter or longer delays after the trial onset; these were adopted from the timing of *Vsynch* and *Vdelay* in an immediately preceding *bimodal exposure* trial. Similarly, the judgment instruction was presented at a similar time compared to the preceding trial, and the spatial offset was defined relative to an assumed movement endpoint that corresponded to the movement error in the preceding trial plus the currently cued movement direction (which was cued although no movement was to be performed).

Experiment 2 comprised three variants of *bimodal judgment* trials. *Double-stimulus* trials were identical to the *bimodal judgment* trials of Experiment 1. In *synchronous-stimulus* trials only *Vsynch* was presented when the outward movement reached the workspace boundary, and in *delayed-stimulus* trials only *Vdelay* was presented during the backward movement.

### Experimental designs

Experiment 1 comprised an initial practice block of 20 trials, a first part to assess integration, and a second part to assess recalibration. Integration was tested in 180 *bimodal judgment* trials. These were 2 repetitions of 90 unique trials: 3 types of position judgments (hand, *Vsynch*, *Vdelay*), 6 pairs of spatial offsets of *Vsynch* and *Vdelay* (− 10°/10°, − 6°/6°, − 2°/2°, 2°/− 2°, 6°/− 6°, 10°/− 10°), 5 cued ranges of target directions (centred on 60°, 75°, 90°, 105°, or 120°). The 90 trials were presented in two blocks of 90 trials each, the trial order being randomized within each block. Recalibration was tested in 300 trials. Initially there was a pre-test phase of 60 trials, 30 *bimodal exposure* trials alternating with 30 *unimodal judgment* trials (10 hand, 10 *Vsynch*, and 10 *Vdelay* in semi-randomized order). This was followed by the exposure phase of 180 *bimodal exposure* trials that varied only in the cued target direction. Half of the participants were exposed to *Vsynch* and *Vdelay* at + 30° and − 30°, for the other half this was reversed. Finally, there was a post-test phase of 60 trials. The order of the unimodal trials in the pre-test and post-test was randomized. Trials were arranged in three blocks of 90 trials (pre-test and exposure), 120 trials (exposure), and 90 trials (exposure and post-test), which were of approximately equal durations.

Experiment 2 comprised an initial practice block of 20 trials followed by 420 experimental trials. These were 2 repetitions of 210 unique trials: 90 were *double-stimulus* trials, 60 *synchronous-stimulus* trials, and 60 *delayed-stimulus* trials. These 90 or 60 trials varied in the 3 or 2 types of position judgments (hand, *Vsynch*, and/or *Vdelay*), the 6 pairs of spatial offsets of *Vsynch* and *Vdelay* (− 10°/10°, − 6°/6°, − 2°/2°, 2°/− 2°, 6°/− 6°, 10°/− 10°), and the 5 cued ranges of target directions (60°, 75°, 90°, 105°, or 120°). The 420 trials were presented in six blocks of 70 trials each, trial order being randomized within each set of 210 trials.

### Trial sequence

Trials were composed of two or three phases: preparation, stimulus exposure, and judgment. In the preparation phase (Fig. 4a) an arrow directed participants to an initial position, which was randomly selected within a rectangular area just below (− 20 to − 40 mm) and to both sides (− 15 to + 15 mm) of the centre of the semi-circular workspace. One second after reaching the initial position, a WiFi-like symbol was presented for one second and cued one of the five potential ranges of directions for the outward-movement. These symbols were also presented in *unimodal judgment* trials in which no movements were performed because they also indicated a range of directions in which visual stimuli were likely to appear. Subsequently, the start position in the centre of the workspace was marked by an outline circle (7 mm diameter) and the position of the hand relative to the start position was indicated by a filled circle (6 mm diameter). Once the start position was reached and the stylus remained for 500 ms within a tolerance range of 2.5 mm from its centre, the circles disappeared and an auditory beep (0.3 s) signalled the go for the outward movement. The hand movement from the initial position to the start position was included to force participants’ eyes off a target location for their outward movement. The colour of the outline circle indicated the current type of trial. It was light grey in all bimodal trials (i.e., *bimodal judgment* and *bimodal exposure*). In *unimodal judgment* trials without movement it was red, instructing participants to stay at the start position, and in *unimodal judgment* trials with movement it was green.

The preparation phase of each trial was followed by the exposure phase (Fig. 4b). The beginning of the outward movement was recorded when the distance from the start position exceeded 2.5 mm, and its end was defined when 97% of the 150 mm distance to the workspace boundary were passed. The movement reversal was defined as the time period from passing the 97% threshold in the outward direction until passing that threshold in the backward direction. The backward movement started when this threshold was passed and ended (signalled by a short beep) when the position of the hand had not changed by more than 2.5 mm for 500 ms. In *bimodal judgment* and *bimodal exposure* trials, *Vsynch* and *Vdelay* were presented at the appropriate times, and in *unimodal judgment* trials only a movement was performed or only a visual stimulus was presented with its timing and position as described above.

During the judgment phase (Fig. 4c) of *bimodal* and *unimodal judgment* trials, the required judgment was instructed upon completion of the backward movement and after the visual stimuli had been presented. (In *unimodal judgment* trials without a movement the timing was adopted from the preceding *bimodal exposure* trial.) A short text was presented on the monitor below the start position, ‘hand’, ‘yellow cloud’ or ‘blue cloud’, and remained visible until the end of the judgment. One second after the instruction, a light grey filled circular marker (2 mm diameter) appeared at a random position on the invisible semi-circle that corresponded to the workspace boundary. Participants could navigate the marker along the semi-circular path by way of small movements of the stylus to the left and right relative to its position at the end of the backward movement (or the start position when no movement had been performed). When the position of the marker matched the remembered hand endpoint or the remembered position of the instructed visual stimulus, whichever had to be judged, participants pressed the button on the stylus to confirm their judgment. There were no time constraints for this.

### Data pre-processing

As in previous studies (e.g.,^32^), we screened the judgment errors for outliers and the movement trajectories for deviations from the instructions, which required smooth outward movements in the cued direction followed by immediate backward movements. The following criteria were used to detect outliers (in brackets the percentages of discarded trials are indicated separately for judgment trials and exposure trials): (1) the absolute angular deviation between the physical and judged positions of a visual stimulus or the movement endpoint of the hand was larger than 20° (judgment trials Exp.1, Exp. 2: 3.29%, 5.88%), (2) the outward movement included a reversal of more than 10 mm (judgment trials Exp.1, Exp. 2: 0.69%, 0.45%; exposure trials Exp. 1: 1.17%). (3) the stylus slid along the workspace boundary for more than 2.5° during movement reversal (judgment trials Exp.1, Exp. 2: 3.23%, 3.41%; exposure trials Exp. 1: 5.17%), (4) the movement direction deviated more than 30° from the centre of the instructed range of movement directions (judgment trials Exp.1, Exp. 2: 2.13%, 3.17%; exposure trials Exp. 1: 2.54%), and (5) the hand moved away from the start position by more than 15 mm in trials in which no movement was to be performed (judgment trials Exp. 1: 0.46%). For Experiment 1, the medians of the number of included trials per participant were (ranges are in brackets): *bimodal judgment* trials with hand position judged: 56.5 (35–60), with *Vsynch* position judged: 55.5 (41–60), and with *Vdelay* position judged: 56.5 (34–60); *unimodal judgment* trials with hand position judged: 17.5 (8–20), with *Vsynch* position judged: 19.0 (17–20), and with *Vdelay* position judged: 19.0 (18–20). The five participants who were replaced by new participants had more than 60% discarded trials in at least one of the different types of judgment trials; three of them made many movements that did not obey the instructions, and two of them made many large errors in judging the hand position. For Experiment 2, the median (and the minimum–maximum) of the number of retained trials for the 7 different types per participant were: *double-stimulus* trials with judgments of hand position: 53.5 (33–59), of *Vsynch*: 50.0 (29–60), and of *Vdelay*: 54.0 (37–59); *synchronous-stimulus* trials with judgments of hand: 53.0 (35–59) and of *Vsynch*: 53.0 (35–58); *delayed-stimulus* trials with judgments of hand: 54.0 (38–59) and of *Vdelay*: 56.0 (39–60).

### Dependent variables to quantify integration

Our primary interest was the proprioceptive bias, that is, the bias of the judged hand position towards the position of *Vsynch*. As the spatial offsets of *Vsynch* and *Vdelay* were symmetric around the movement endpoint, any bias of the hand position towards *Vsynch* was also a bias away from *Vdelay*. In additional explorative analyses or sanity checks, we quantified i) the visual biases of the judged positions of both visual stimuli towards the position of the hand, ii) the consistent judgment errors that were independent of the spatial offsets between visual stimuli and hand position, and iii) the intra-individual variability of the judgments. As the timing of the events in each trial depended on the precise movement kinematics, we also quantified the durations of individual phases of the experimental trials, such as between the start signal for the movement and the presentation of *Vsynch* or *Vdelay*, or the duration of the judgments.

Biases, consistent judgment errors, and judgment variability were determined from regression analyses performed for each participant and judgment type separately. Specifically, for each *bimodal judgment* trial we computed the judgment error, that is, the deviation of the judged from the physical angular position of the hand or a visual stimulus (positive errors were counter clockwise, negative errors were clockwise). These judgment errors were regressed on the angular spatial offsets, i.e. the difference between the physical angular positions of visual stimulus and hand endpoint. The slope of this regression provides a measure of the proportional bias, that is, of the judgment error as a proportion of the angular offset. The intercept reflects consistent judgment errors independent of the relative positions of the visual stimuli, and the standard deviation of the residuals can serve as measure of intra-individual judgment variability. Figure 2a shows an example for an individual participant’s judgments of hand positions.

### Dependent variables to quantify recalibration

Recalibration was quantified based on the *unimodal judgment* trials during pre-test and post-test. We report the mean judgment errors during the pre-test and the recalibration biases. The latter were computed as differences between the judgment errors in post-test and pre-test with the sign of the differences chosen such that positive differences are adaptive: for hand judgments a positive bias represents a shift towards the position of *Vsynch* in the preceding *bimodal exposure* trials; for visual judgments a positive bias represents a shift towards the hand position in the preceding *bimodal exposure* trials. Motor adaptation was assessed as the change of the movement directions in *unimodal judgment* trials from pre-test to post-test. Positive values are adaptive: they represent a shift of the movement direction away from the position of *Vsynch* in the preceding *bimodal exposure* trials.

### Statistical analyses

For both experiments we tested the regression slopes and intercepts against zero using two-sided t-tests. We further compared the biases between Experiments 1 and 2 using independent-samples t-tests and tested them against zero for the combined sample. For recalibration the adaptive biases in judgments and movement directions were tested against zero using t-tests as well. For the main purpose of Experiment 2, the following two analyses were implemented. First, we compared the proportional biases of the judged hand position towards *Vsynch* in *double-stimulus* and *synchronous-stimulus* trials. Second, the proportional bias of the judged hand position towards *Vsynch* in *double-stimulus* trials was compared with the sum of the biases in *synchronous-stimulus* and *delayed-stimulus* trials. As effect size for these tests we report d_z_, which is the ratio of mean and standard deviation of the measurements (for paired-sample t-tests the mean and standard deviation of the differences are entered). Judgment variability was compared using an ANOVA with the within-participant factors judgment type and pre-test vs post-test (for recalibration). Corresponding ANOVAs served to analyse the durations of various trial phases. ANOVA results are reported with Greenhouse–Geisser epsilon and adjusted *p*. As effect size we report partial eta-squared, $\eta_{p}^{2}$, the ratio of the sum of squares for an effect divided by the sum of the sums of squares for the effect and the error. While we overall report a larger number of statistical tests, we emphasize that the main questions and hypotheses were addressed by the following four tests: those on the proprioceptive (hand) bias for integration and recalibration in Experiment 1, and the comparison of the proprioceptive integration bias between double- and single-stimulus conditions in Experiment 2.

## Ethical approval

All procedures were approved by the Bielefeld University Ethics Committee and in accordance with the 1964 Helsinki declaration. Informed consent was obtained from all individual participants included in the study.

## Data Availability

All data analysed in this study are available at https://pub.uni-bielefeld.de/record/2954727.
